# Circulating tumor DNA and prognosis in gastric cancer patients undergoing surgery: meta-analysis and trial sequential analysis

**DOI:** 10.3389/fonc.2026.1774979

**Published:** 2026-05-18

**Authors:** Jidong Bian, Jinchao Liu

**Affiliations:** Department of Gastrointestinal Surgery, Zibo Municipal Hospital, Zibo, Shandong, China

**Keywords:** circulating tumor DNA, gastric cancer, meta‐analysis, prognosis, trial sequential analysis

## Abstract

**Background:**

Circulating tumor DNA (ctDNA) is a promising biomarker for monitoring minimal residual disease (MRD) and recurrence risk in gastric cancer. Prior meta-analyses were limited by heterogeneous cohorts, inconsistent HR extraction, and lack of trial sequential analysis (TSA). This study provides an updated, methodologically rigorous synthesis focusing exclusively on resectable gastric cancer.

**Methods:**

A systematic search of four major databases was performed to May 2025 (PROSPERO: CRD42025107578). Eligible studies included surgically treated gastric cancer patients with perioperative ctDNA assessment and reported hazard ratios (HRs) for overall survival (OS) or disease-free survival (DFS). Multivariate Cox HRs were prioritized, with one HR extracted per time window to ensure independence. Random-effects models, subgroup and sensitivity analyses, and publication bias assessments were conducted in R 4.5.0. TSA used O’Brien–Fleming monitoring boundaries to evaluate evidence sufficiency.

**Results:**

Twenty-five studies (60 datasets) were included. ctDNA positivity was associated with significantly worse OS (HR = 2.31, 95% CI 1.78–3.00) and DFS (HR = 2.36, 95% CI 1.69–3.29). Subgroup analyses showed consistent effects across detection platforms, sample sources, regions, and stages. Post-operative ctDNA demonstrated stronger prognostic value than pre-operative measurements for both OS (HR = 3.47 vs. 1.99) and DFS (HR = 4.14 vs. 1.97). Results were robust in sensitivity analyses. Publication bias was present for OS but did not materially alter pooled estimates. TSA showed that cumulative Z-curves crossed the O’Brien–Fleming boundaries and met or exceeded the required information size.

**Conclusion:**

ctDNA positivity reliably predicts poorer postoperative survival in resectable gastric cancer, with post-operative ctDNA providing the strongest prognostic signal. TSA confirms that current evidence is sufficiently conclusive to support clinical relevance.

**Systematic review registration:**

https://www.crd.york.ac.uk/PROSPERO/, identifier CRD42025107578.

## Introduction

Gastric cancer (GC) remains a major global health challenge and is a leading cause of cancer-related mortality (1, 2). For patients with resectable disease, curative-intent gastrectomy is the primary treatment modality (3). However, despite technically successful R0 resection, many patients still experience recurrence due to minimal residual disease (MRD) that escapes routine detection (4). Circulating tumor DNA (ctDNA), a tumor-derived component of cell-free DNA, has emerged as a promising non-invasive biomarker capable of reflecting tumor burden, molecular evolution, and postoperative MRD (5, 6). Early evidence suggests that both pre-operative and post-operative ctDNA may possess prognostic value for disease-free survival (DFS) and overall survival (OS) in patients undergoing surgery (7, 8).

Previous meta-analyses have attempted to evaluate the prognostic significance of ctDNA in gastric cancer, but their reliability is limited by several methodological shortcomings. Many pooled heterogeneous patient populations—including individuals receiving chemotherapy, immunotherapy, or palliative treatment—together with surgically treated cohorts, thereby conflating fundamentally different disease trajectories (9, 10). Others extracted univariate hazard ratios despite the availability of multivariate Cox estimates, or included multiple perioperative time points from the same cohort, violating statistical independence (11). In addition, earlier reviews occasionally incorporated misclassified or inaccurate data, which may have introduced bias into pooled estimates. These issues highlight the need for a focused reassessment specifically restricted to patients undergoing curative-intent surgery, where survival outcomes directly reflect recurrence risk and ctDNA is most clinically relevant for MRD detection.

To address these limitations, the present study incorporates updated literature, applies rigorous and standardized data extraction rules (12), and ensures that each cohort contributes only one hazard ratio per time window, with multivariate estimates prioritized whenever available. Importantly, this work also integrates trial sequential analysis (TSA) to evaluate the sufficiency and robustness of the cumulative evidence—an analytic step not undertaken in previous reviews (13, 14). By correcting prior inconsistencies, synthesizing newly published data, and applying TSA, this meta-analysis aims to provide the most accurate contemporary assessment of the prognostic value of ctDNA in gastric cancer patients undergoing curative-intent surgery.

## Materials and methods

### Literature search

This systematic review and meta-analysis was conducted in accordance with PRISMA guidelines (12) and registered in PROSPERO (CRD42025107578). PubMed, Embase, Web of Science, and the Cochrane Library were searched from inception to May 2025 using terms related to gastric cancer, circulating tumor DNA, cell-free DNA, methylation, prognosis, and survival. Reference lists of relevant publications and reviews were screened to identify additional eligible studies.

### Eligibility criteria

Eligible studies met the following criteria: (1) included patients with operable or resectable gastric cancer (typically stages I–III) who underwent curative-intent surgery; (2) assessed ctDNA in plasma or serum using PCR-based or sequencing-based platforms; (3) reported hazard ratios (HRs) for DFS or OS; and (4) were prospective or retrospective cohort studies. Mixed stage I–IV cohorts were included only when all patients were clearly reported to have undergone surgery and postoperative follow-up. Because operation type and metastatic status were not uniformly reported in the original studies, these variables could not be summarized in a fully standardized manner across all cohorts. DFS was defined as the interval from surgery to recurrence or death; studies reporting recurrence-free survival (RFS) or progression-free survival (PFS) using equivalent definitions were treated as reporting DFS (15). Studies involving unresectable/metastatic disease, those analyzing tumor tissue rather than circulating cfDNA, or those lacking extractable HRs were excluded. When duplicate cohorts existed, the most complete dataset was used.

### Data extraction

Two investigators independently extracted study characteristics, ctDNA assay type, sample source, timing of ctDNA sampling, and HRs for DFS and OS. Following the Cochrane Handbook (Chapter 10), multivariate Cox regression HRs were prioritized over univariate Kaplan–Meier estimates when both were available. For studies reporting multiple ctDNA time points (baseline, post-neoadjuvant therapy, postoperative, or post–adjuvant chemotherapy), only one HR per time window (pre-operative and post-operative) was extracted to ensure statistical independence. For postoperative analyses, the earliest sampling window (16, 17) (approximately 4–8 weeks after surgery and before adjuvant therapy) was prioritized to maximize comparability and reflect MRD. Each study contributed at most one HR for DFS and one for OS per time window. HRs unavailable in tabular form were reconstructed from Kaplan–Meier curves using validated methods (18).

### Quality assessment

Study methodological quality was evaluated using the Newcastle–Ottawa Scale (NOS), assessing selection, comparability, and outcome domains (Wells et al., Ottawa Hospital Research Institute). Studies scoring ≥6 were considered high quality. Any discrepancies between reviewers were resolved through discussion until consensus was reached.

### Outcomes

The primary outcome was DFS, defined as the time from curative-intent surgery to recurrence or death. Because several studies reported RFS or PFS using identical definitions, these endpoints were treated as equivalent to DFS (15). The secondary outcome was OS. For each study, only one DFS HR and one OS HR were extracted for each perioperative time window (pre-operative and post-operative) to avoid duplication and maintain independence.

### Statistical analysis

All statistical analyses were performed using R software (version 4.5.0). Pooled HRs and 95% confidence intervals (CIs) were calculated with a random-effects model. Heterogeneity was quantified using Cochran’s Q test and the I² statistic. Prespecified subgroup analyses were conducted based on geographic region, ctDNA assay platform, sample source, pathological stage, sample size, and timing of ctDNA assessment (pre-operative vs. post-operative). Sensitivity analyses were performed by sequentially excluding individual studies. Publication bias was evaluated using funnel plots, Egger’s test, and the trim-and-fill procedure, all implemented in R (packages meta, metafor, and dmetar). Forest plots, funnel plots, subgroup analyses, and sensitivity analyses were generated entirely in R 4.5.0.

### Trial sequential analysis

TSA was conducted using R software (version 4.5.0) based on log(HR) and its standard error (SE). The O’Brien–Fleming α-spending function was applied to construct sequential monitoring boundaries and estimate the required information size (RIS), assuming a two-sided α of 0.05 and 80% power (19, 20). TSA curves, RIS calculations, and monitoring boundaries were generated using dedicated R scripts based on established TSA theoretical frameworks. Because TSA requires statistical independence, each study contributed only one HR per survival endpoint. For studies reporting multiple perioperative ctDNA time points, the earliest postoperative HR (4–8 weeks after surgery, before adjuvant therapy) was prioritized; when postoperative data were unavailable, the most comparable perioperative HR was used.

## Results

### Study characteristics

**Figure 1:**
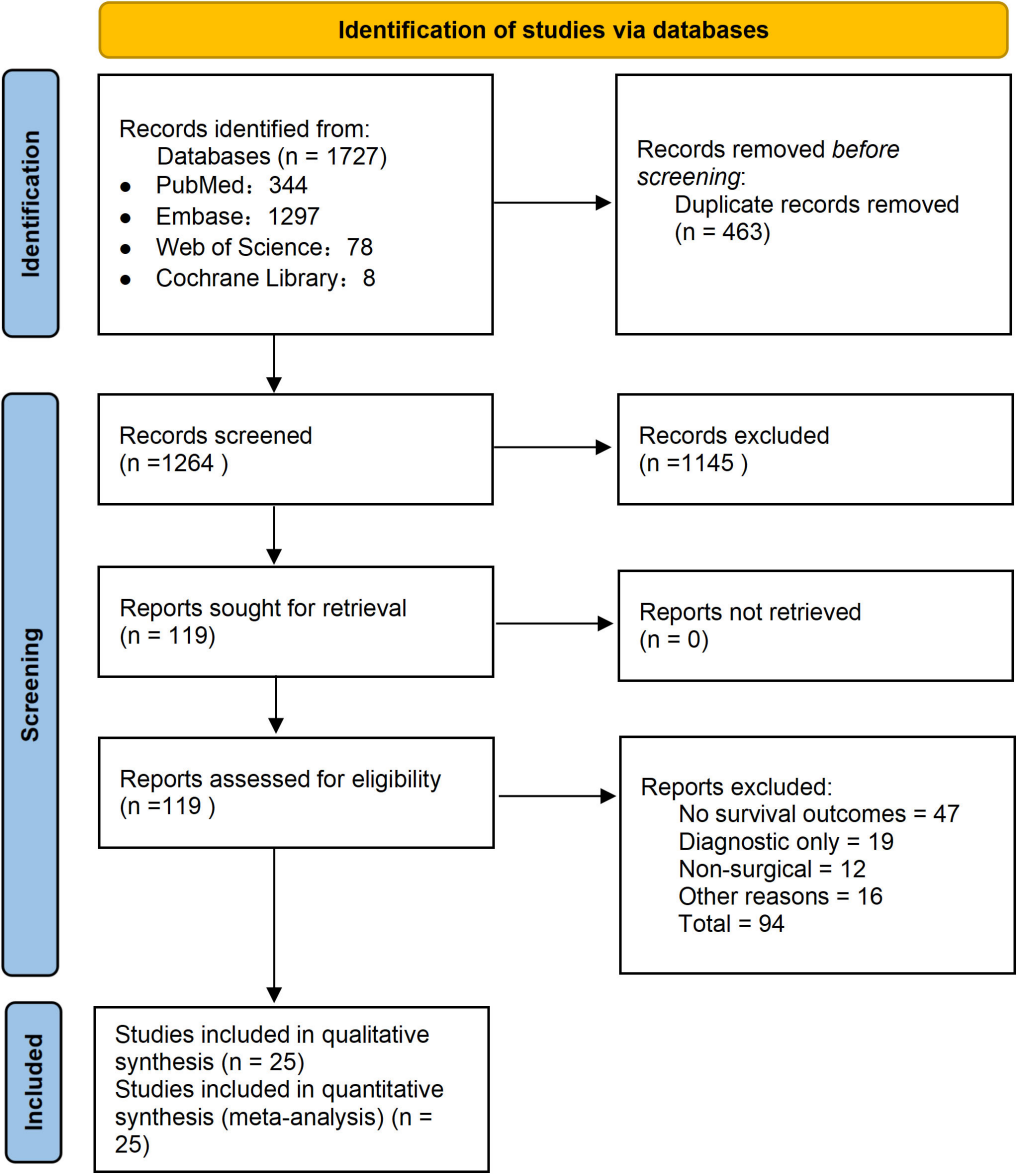
PRISMA flow diagram of study selection.

A total of 1727 records were identified through database searching and manual cross-referencing. After removing duplicates and screening titles and abstracts, 119 full-text articles were assessed, and 25 studies were finally included in the quantitative synthesis (**Figure 1**). These studies were published between 2013 and 2025 and collectively contributed 60 ctDNA datasets for OS and DFS (7, 8, 21–43).

ctDNA was measured using PCR-based methylation assays or next-generation sequencing platforms. Plasma was the predominant sample source, whereas several studies used serum. Most studies assessed ctDNA at a single perioperative time point; when multiple time points were reported, only one HR per time window was included to preserve statistical independence. The detailed search strategy, study-level characteristics, and HR extraction procedures—including HRs digitized from Kaplan–Meier curves—are provided in **Supplementary Table 1**-**S3**.

### Quality of included studies

Methodological quality assessed by the Newcastle–Ottawa Scale (NOS) ranged from 6 to 9 points, indicating overall moderate-to-high study quality. Most studies demonstrated adequate control of confounding through multivariate Cox analyses, clear survival definitions, and sufficient follow-up. No study was excluded based on quality concerns (**Supplementary Table 4**).

### Overall effect on survival outcomes

**Figure 2:**
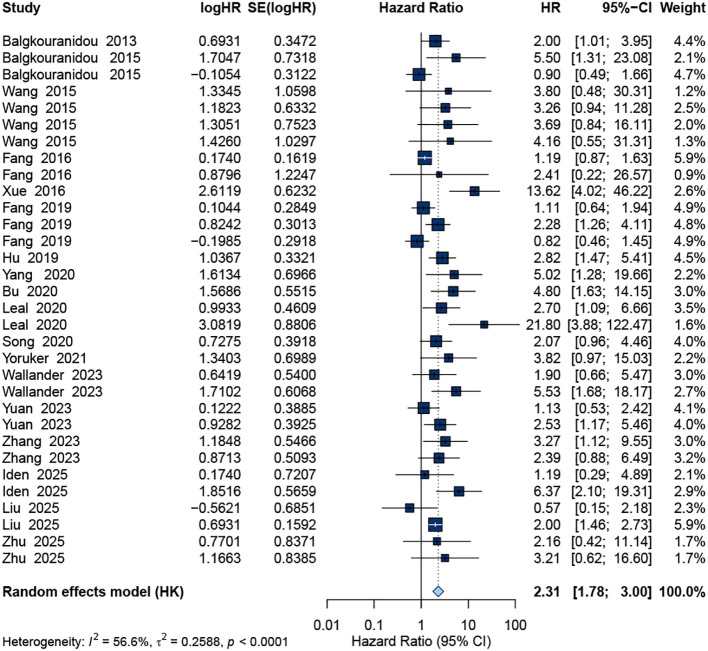
Forest plot of the pooled HR for OS.

A total of 32 study datasets contributed to the pooled analysis of OS. As shown in **Figure 2**, ctDNA positivity was significantly associated with an increased risk of mortality. The random-effects model yielded a pooled HR of 2.31 (95% CI: 1.78–3.00), indicating that patients with detectable ctDNA had more than a twofold higher risk of death compared with ctDNA-negative patients. Moderate heterogeneity was observed across studies (I² = 56.6%, *p* < 0.0001).

**Figure 3:**
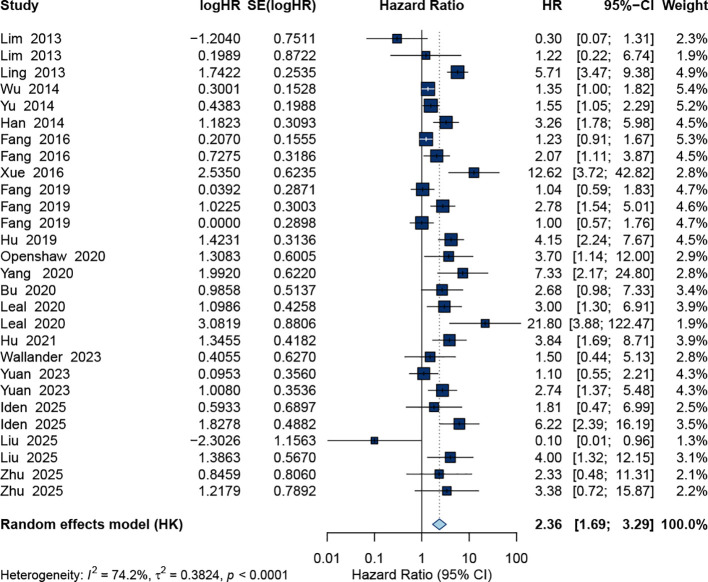
Forest plot of the pooled HR for DFS.

For DFS, 28 datasets were included. The pooled estimate demonstrated that ctDNA positivity was also significantly associated with worse DFS. The combined HR was 2.36 (95% CI: 1.69–3.29) under a random-effects model (**Figure 3**). Heterogeneity was substantial (I² = 74.2%, *p* < 0.0001), reflecting diversity in ctDNA detection methods, sampling time points, and patient characteristics.

Together, these results indicate that across all included studies, ctDNA positivity consistently correlated with both inferior OS and shorter DFS in patients undergoing curative-intent surgery for gastric cancer.

### Subgroup analysis

Subgroup analyses were conducted to explore potential sources of heterogeneity and to assess the consistency of the prognostic impact of ctDNA across clinically relevant strata (**Table 1**, **2**). For OS, the adverse effect of ctDNA positivity was observed in both Asian (pooled HR = 2.11, 95% CI 1.57–2.83; I² = 53.9%) and non-Asian cohorts (HR = 2.89, 95% CI 1.54–5.40; I² = 62.6%), with no evidence of a between-region difference (*p* for subgroup difference = 0.312). Similar effect sizes were seen when studies were stratified by detection method and sample source: PCR-based assays (HR = 2.60, 95% CI 1.68–4.01) and sequencing-based assays (HR = 2.03, 95% CI 1.41–2.91) both showed significant associations, as did plasma-based (HR = 2.11, 95% CI 1.57–2.85) and serum-based (HR = 3.00, 95% CI 1.63–5.53) measurements, without statistically significant interaction by method or source (*p* for subgroup difference = 0.264 and 0.247, respectively). The effect of ctDNA also persisted irrespective of study size (≥50 vs. <50 patients).

**Table 1:** Subgroup analyses of pooled HRs for OS and heterogeneity.

Subgroup	No. of study datasets	Q test	Pooled HR (95% CI)	Heterogeneity	
				P	I²
Country/region		0.312			
Asia	22		2.11(1.57-2.83)	0.002	53.90%
Non-Asia	10		2.89(1.54-5.40)	0.004	62.60%
Detection methods		0.264			
PCR-based	14		2.60(1.68-4.01)	< 0.001	63.70%
Sequencing-based	17		2.03(1.412-2.91)	0.008	50.90%
Sample source		0.247			
Plasma	23		2.11(1.57-2.85)	0.001	55.00%
Serum	9		3.00(1.63-5.53)	0.009	60.80%
Time of sample collection		0.043			
Pre-operatively	24		1.99(1.48-2.66)	< 0.001	53.60%
Post-operatively	8		3.47(1.98-6.10)	0.060	48.30%
Study size		0.280			
≥50	22		2.13(1.55-2.93)	< 0.001	61.80%
<50	10		2.85(1.74-4.67)	0.197	26.70%
Stage		0.015			
I–III (early stage)	18		1.80(1.32-2.46)	0.013	48.20%
I–IV (all stages)	14		3.29(2.14-5.04)	< 0.001	64.70%

HR, hazard ratio; CI, confidence interval; PCR, polymerase chain reaction.

**Table 2:** Subgroup analyses of pooled HRs for DFS and heterogeneity.

Subgroup	No. of study datasets	Q test	Pooled HR (95% CI)	Heterogeneity	
				P	I² (%)
Country/region		0.157			
Asia	22		2.14(1.47-3.12)	< 0.0001	76.30%
Non-Asia	6		3.65(1.56-8.50)	0.127	41.60%
Detection methods		0.881			
PCR-based	12		2.52(1.42-4.47)	< 0.0001	83.40%
Sequencing-based	15		2.18(1.34-3.53)	< 0.001	63.30%
Sample source		0.648			
Plasma	19		2.19(1.48-3.25)	< 0.0001	65.00%
Serum	9		2.60(1.23-5.49)	< 0.0001	84.10%
Time of sample collection		0.011			
Pre-operatively	20		1.97(1.35-2.89)	< 0.0001	76.50%
Post-operatively	8		4.14(2.43-7.07)	0.288	17.90%
Study size		0.167			
≥50	19		2.13(1.40-3.23)	< 0.0001	79.60%
<50	9		3.36(1.83-6.17)	0.250	21.70%
Stage		0.504			
I–III (early stage)	12		2.06(1.17-3.65)	< 0.001	67.30%
I–IV (all stages)	16		2.58(1.64-4.06)	< 0.0001	78.80%

HR, hazard ratio; CI, confidence interval; PCR, polymerase chain reaction.

**Figure 4:**
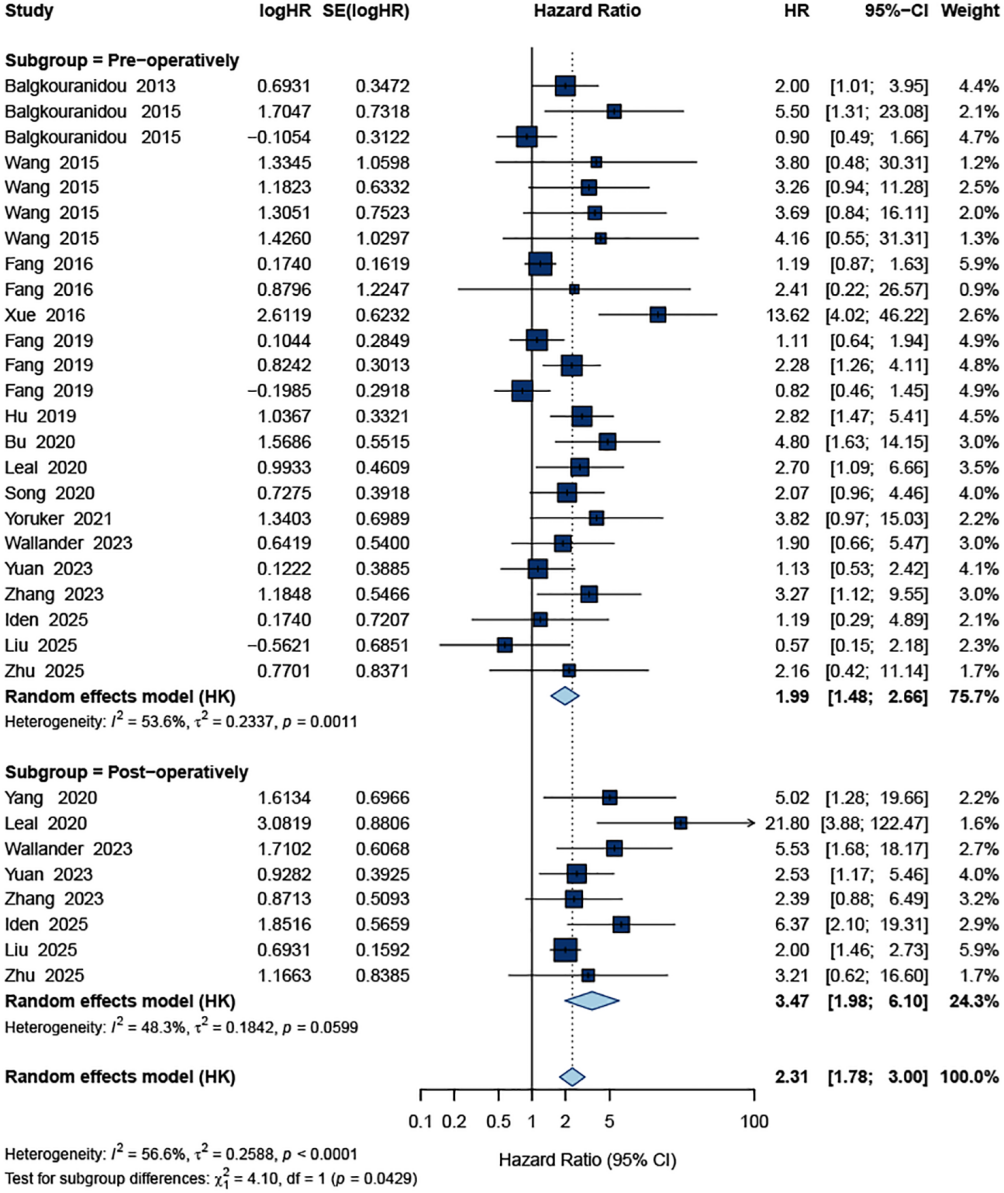
Subgroup forest plot of the pooled HR for OS based on ctDNA measured pre-operatively and post-operatively.

**Figure 5:**
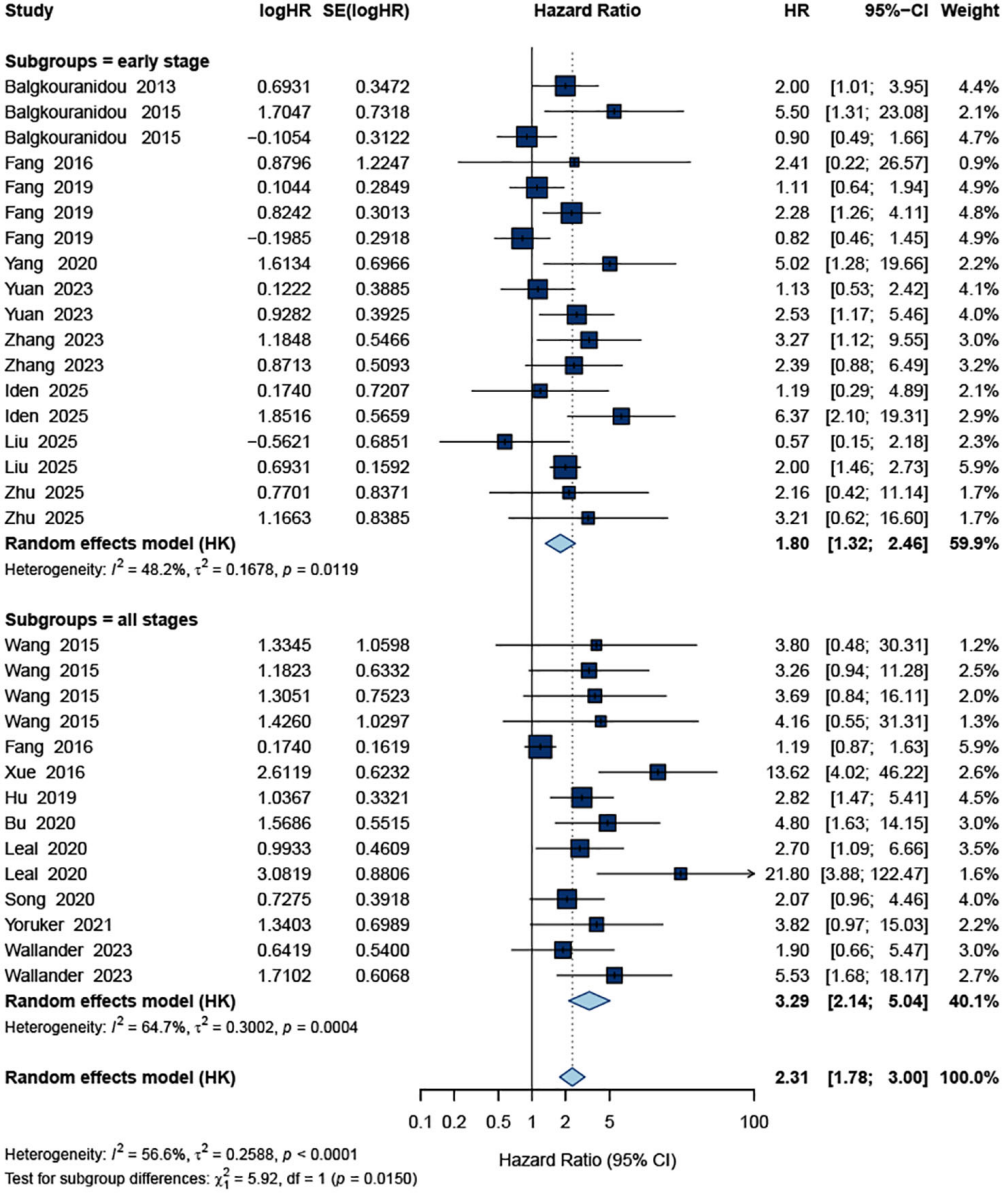
Subgroup forest plot of the pooled HR for OS according to pathological stage.

When stratified by timing of ctDNA assessment, a clear gradient emerged for OS. Pre-operative ctDNA positivity was associated with an increased risk of death (HR = 1.99, 95% CI 1.48–2.66; I² = 53.6%), whereas post-operative ctDNA positivity showed an even stronger association (HR = 3.47, 95% CI 1.98–6.10; I² = 48.3%). The test for subgroup difference was statistically significant (*p* = 0.043), indicating that post-operative ctDNA provides greater prognostic discrimination than pre-operative assessment (**Figure 4**). In addition, for OS the pooled effect was stronger in studies including all stages (I–IV; HR = 3.29, 95% CI 2.14–5.04; I² = 64.7%) than in early-stage (I–III) cohorts (HR = 1.80, 95% CI 1.32–2.46; I² = 48.2%), with a significant interaction by stage (*p* = 0.015; **Figure 5**).

The patterns for DFS were broadly consistent with those for OS (**Table 2**). The association between ctDNA positivity and shorter DFS was observed in both Asian (HR = 2.14, 95% CI 1.47–3.12; I² = 76.3%) and non-Asian studies (HR = 3.65, 95% CI 1.56–8.50; I² = 41.6%), with no significant between-region difference (*p* = 0.157). Stratification by detection platform (PCR-based vs. sequencing-based), sample source (plasma vs. serum), study size (≥50 vs. <50), and stage (I–III vs. I–IV) all yielded consistently elevated HRs without statistically significant subgroup effects.

**Figure 6:**
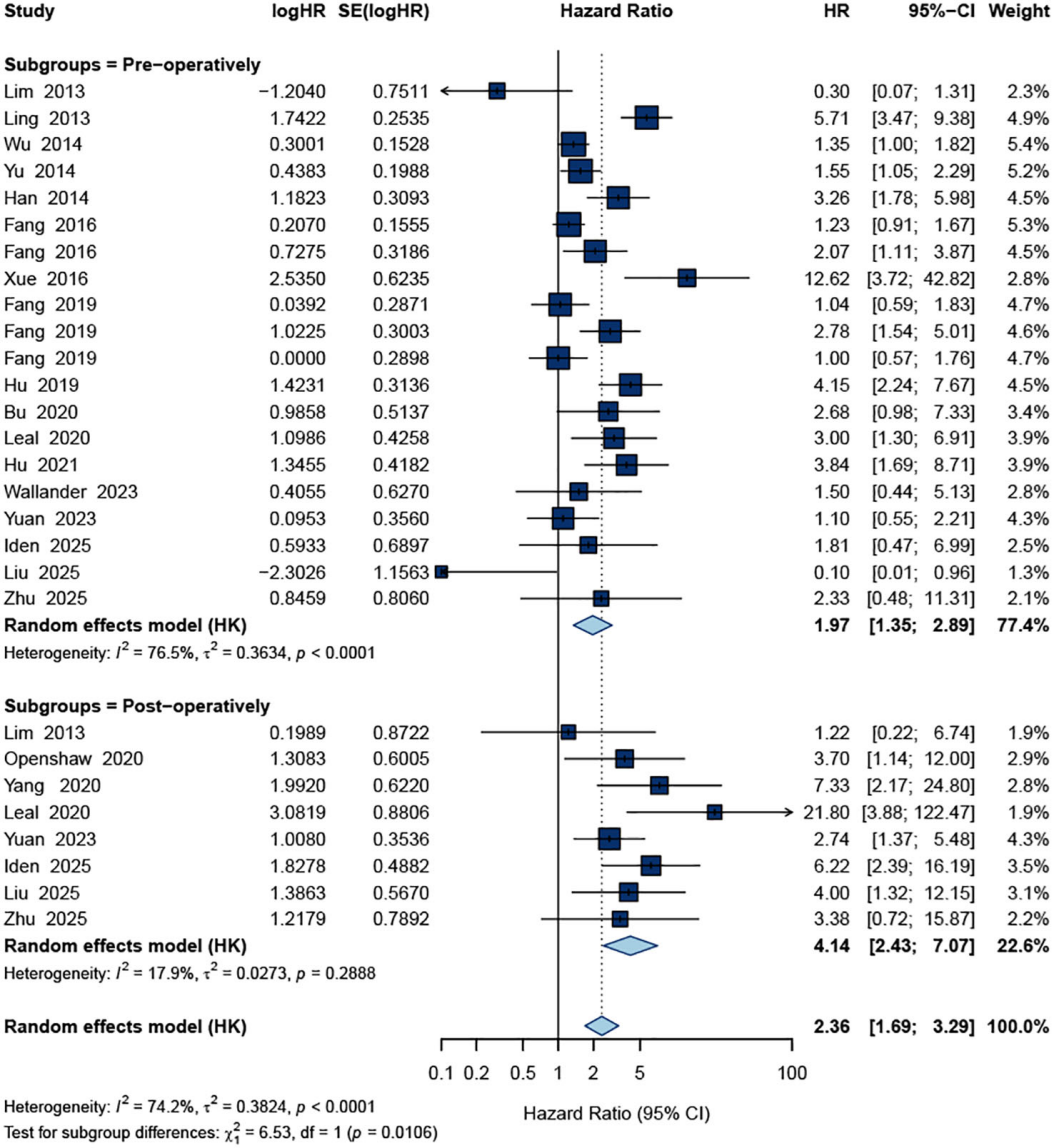
Subgroup forest plot of the pooled HR for DFS based on ctDNA measured pre-operatively and post-operatively.

By contrast, the timing of ctDNA sampling again showed a marked difference for DFS. Pre-operative ctDNA positivity was associated with poorer DFS (HR = 1.97, 95% CI 1.35–2.89; I² = 76.5%), while post-operative ctDNA positivity was associated with an even higher risk (HR = 4.14, 95% CI 2.43–7.07; I² = 17.9%). The interaction between pre-operative and post-operative subgroups was significant (*p* = 0.011; **Figure 6**), supporting a stronger prognostic value of post-operative ctDNA, consistent with its role as a marker of minimal residual disease.

### Publication bias

**Figure 7:**
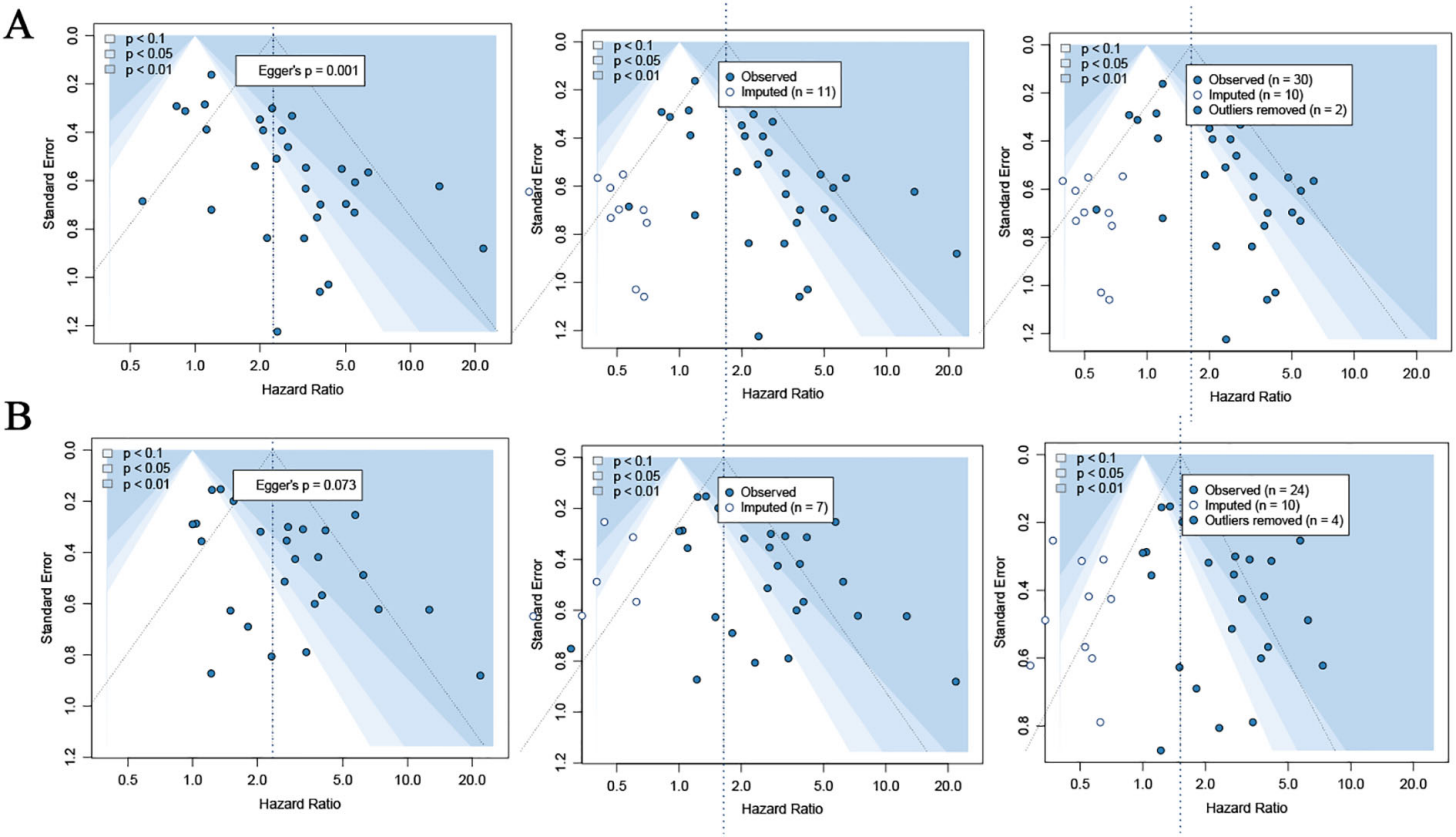
Funnel plots assessing publication bias for OS and DFS. **(A)** Funnel plots for OS, including the original plot, trim-and-fill imputation, and outlier-adjusted plot. Egger’s test indicated significant asymmetry (*p* = 0.001). **(B)** Funnel plots for DFS, including the original plot, trim-and-fill imputation, and outlier-adjusted plot. Egger’s test showed no significant asymmetry (*p* = 0.073).

Publication bias was evaluated using funnel plot symmetry, Egger’s regression test, and the trim-and-fill method (**Figure 7**).

For OS (**Figure 7A**), visual inspection of the funnel plot suggested noticeable asymmetry, and Egger’s test confirmed significant small-study effects (*p* = 0.001). Application of the trim-and-fill procedure imputed 11 potentially missing studies (middle panel), after which the adjusted funnel plot remained asymmetric but the pooled effect estimate continued to indicate a statistically significant association between ctDNA positivity and worse OS. Outlier-adjusted plots (right panel) also demonstrated persistent asymmetry despite removal of extreme points, suggesting that publication bias may be present but does not fully account for the observed survival associations.

For DFS (**Figure 7B**), the funnel plot appeared more symmetric, and Egger’s test showed no statistically significant evidence of publication bias (*p* = 0.073). The trim-and-fill method imputed 7 missing studies, and the adjusted effect estimate remained largely unchanged. Outlier-adjusted funnel plots also did not demonstrate meaningful deviation from the primary analysis, indicating that publication bias is unlikely to materially influence the DFS findings.

Taken together, these analyses indicate that although publication bias may exist for OS, the effect estimates are robust to correction, and there is no significant evidence of publication bias for DFS.

### Sensitivity analysis

Sensitivity analyses were performed using a leave-one-out approach to assess the stability of the pooled estimates. Sequential exclusion of individual studies did not materially change the overall effect sizes for either OS or DFS, indicating that no single study disproportionately influenced the pooled hazard ratios. The direction and magnitude of the associations remained consistent across iterations, supporting the robustness of the primary findings.

For both OS and DFS, outlier-adjusted funnel plots (**Figures 7A, B**, right panels) showed minimal change in pooled effect estimates after statistically identified outliers were removed, further reinforcing the stability of the results. These findings demonstrate that the prognostic association between ctDNA positivity and adverse survival outcomes was not driven by small-study effects or influential outliers.

### Trial sequential analysis results

**Figure 8:**
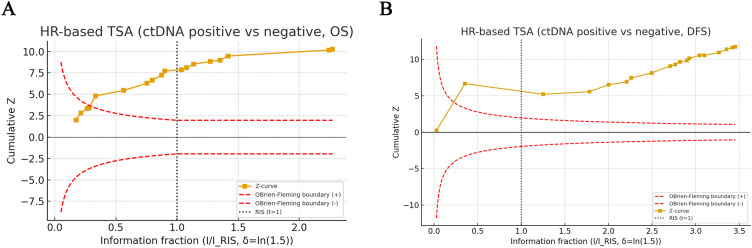
TSA of the association between ctDNA positivity and survival outcomes. **(A)** TSA for OS. **(B)** TSA for DFS. The yellow line represents the cumulative Z-curve. Red dashed lines indicate the O’Brien–Fleming monitoring boundaries. The black dotted vertical line denotes the required information size (RIS).

HR-based trial sequential analysis further evaluated the reliability of the associations between ctDNA positivity and survival outcomes (**Figure 8**). For OS (**Figure 8A**), the cumulative Z-curve crossed the conventional significance boundary and the O’Brien–Fleming monitoring boundary early and remained well above both as additional studies were accrued. The information fraction exceeded the required information size (RIS), suggesting that the cumulative evidence is statistically sufficient to support an association between ctDNA positivity and increased mortality risk, with a reduced risk of random error.

For DFS (**Figure 8B**), the cumulative Z-curve also crossed the O’Brien–Fleming boundary before reaching the RIS and continued to rise as further information accumulated, ultimately lying far beyond both the monitoring boundary and the RIS line. Together, these findings support the statistical robustness of the observed associations. However, TSA does not resolve important sources of uncertainty, including biological and methodological heterogeneity across studies, and therefore these results should not be interpreted as eliminating the need for further prospective validation and ctDNA-guided interventional trials.

## Discussion

In this meta-analysis of 25 studies including 60 ctDNA datasets, we demonstrate that circulating tumor DNA positivity is a consistent and significant predictor of inferior survival outcomes in patients undergoing curative-intent gastrectomy. Beyond the overall pooled associations (OS HR = 2.31; DFS HR = 2.36), several findings from the subgroup analyses provide important additional insights.

Most notably, post-operative ctDNA exhibited the strongest prognostic value, with hazard ratios for both OS and DFS approximately twice those observed for pre-operative ctDNA, consistent with prior prospective studies demonstrating markedly higher recurrence risk in patients with detectable post-operative ctDNA (7, 16, 44, 45). This finding is biologically coherent: whereas pre-operative ctDNA primarily reflects baseline tumor burden, post-operative ctDNA captures MRD—a direct indicator of persistent micrometastatic clones capable of driving early relapse, as supported by fundamental ctDNA biology studies demonstrating that post-surgical ctDNA represents residual viable tumor clones (46, 47). Previous MRD-oriented studies in colorectal, esophageal, and gastric cancers have similarly shown that ctDNA persistence after surgery is one of the earliest and most specific indicators of recurrence, reinforcing that post-operative ctDNA provides substantially greater prognostic discrimination than pre-operative sampling (48, 49).

We also found a stage-dependent gradient, with stronger effect sizes in studies including stage IV patients than in those restricted to stage I–III disease, which should be interpreted with caution in light of the different clinical context of advanced disease. Although this pattern is consistent with previous evidence showing markedly higher ctDNA detectability in advanced-stage tumors (46). This is likely attributable to the higher tumor burden, increased genomic instability, and greater ctDNA shedding typically observed in advanced cancers, as documented in mechanistic studies linking tumor burden with ctDNA release (50). Tumors with metastatic potential have been shown to release greater quantities of fragmented tumor DNA into the bloodstream, a relationship demonstrated in early mechanistic ctDNA studies (47), Accordingly, the larger effect sizes observed in cohorts including stage IV patients may partly reflect these biological differences rather than a directly comparable prognostic signal within a minimal residual disease setting. Therefore, while these findings are informative, analyses including stage IV patients should be regarded as hypothesis-generating, and the primary clinical interpretation of our results should remain focused on stage I–III cohorts undergoing curative-intent surgery.

These associations were remarkably consistent across geographic regions, detection platforms, sample sources, and study sizes, highlighting the robustness of ctDNA as a prognostic biomarker. Moreover, TSA demonstrated that both OS and DFS Z-curves crossed the O’Brien–Fleming monitoring boundaries and reached (or exceeded) the required information size, suggesting that the cumulative evidence is statistically conclusive with respect to random error control. However, these findings should be interpreted within the context of substantial biological and methodological heterogeneity across the included studies. Differences in ctDNA detection platforms, target panels, positivity thresholds, sample types, and timing of blood collection may all influence the observed effect sizes and limit direct comparability between studies. Therefore, although TSA strengthens confidence in the statistical reliability of the pooled associations, it does not obviate the need for further prospective ctDNA-guided interventional trials to determine how these findings can be translated into standardized and clinically actionable strategies.

Several earlier meta-analyses have examined the prognostic role of ctDNA in gastric cancer, but their interpretations have been constrained by notable methodological weaknesses (9). Many included unresectable or metastatic patients, mixed treatment modalities such as immunotherapy or palliative chemotherapy, and frequently accepted multiple perioperative ctDNA measurements from the same patient cohort, thereby violating statistical independence (11). Others relied heavily on univariate HRs extracted from Kaplan–Meier curves despite the availability of adjusted multivariate models.

In contrast, our study addressed these limitations by restricting inclusion to operable, surgically treated cohorts, prioritizing multivariate Cox regression HRs, and applying a strict perioperative time-window extraction strategy to preserve statistical independence. These methodological refinements provide a more clinically interpretable estimate of ctDNA’s prognostic value within the curative surgical setting.

This study has several strengths, including a comprehensive literature search, independent data extraction, and standardized rules for HR selection and perioperative time-window definition. TSA provided an additional assessment of cumulative evidence, although its interpretation remains exploratory in the setting of heterogeneous datasets. Several limitations should also be acknowledged. Most included studies were observational, leaving the possibility of residual confounding despite multivariable adjustment. In addition, substantial heterogeneity existed across ctDNA platforms, genomic targets, positivity thresholds, and sampling schedules; several studies had modest sample sizes; and publication bias was detected for OS, although trim-and-fill correction did not materially alter the pooled estimate. Furthermore, although our analysis primarily focused on operable or resectable patients undergoing surgery, some included studies enrolled mixed stage I–IV cohorts when all patients underwent surgery and postoperative follow-up. Detailed information on operation type and metastatic status was not uniformly reported across the original studies, which may limit interpretation within a strictly curative-intent framework.

These findings highlight ctDNA—particularly postoperative ctDNA—as a highly informative biomarker for identifying patients at high risk of recurrence after gastrectomy. ctDNA has the potential to refine postoperative surveillance strategies, identify patients who may benefit from intensified adjuvant therapy, and guide MRD-directed therapeutic trials. Despite the confirmatory TSA results, standardization remains insufficient for immediate clinical implementation. Future work should focus on large-scale, prospective, multi-center studies with harmonized assay methodologies and predefined sampling intervals, as well as randomized trials evaluating ctDNA-guided adjuvant treatment escalation or de-escalation to establish its utility in routine clinical practice.

## Conclusions

This meta-analysis demonstrates that circulating tumor DNA is a robust prognostic biomarker in resectable gastric cancer, with ctDNA positivity consistently associated with significantly worse overall and disease-free survival. Post-operative ctDNA showed the strongest prognostic value, underscoring its role as a surrogate for minimal residual disease, and the effect remained stable across regions, detection platforms, sample sources, and pathological stages. Trial sequential analysis further confirmed that the cumulative evidence is statistically sufficient and unlikely to be overturned by additional studies. These findings support the clinical potential of ctDNA—particularly early post-operative assessment—for recurrence risk stratification, individualized postoperative surveillance, and MRD-guided adjuvant therapy design, although standardized assay methodologies and prospective, multi-center trials remain necessary before ctDNA can be implemented in routine postoperative management.

## Data Availability

The original contributions presented in the study are included in the article/**Supplementary Material**. Further inquiries can be directed to the corresponding author.
